# *Helicase-like transcription factor* (*Hltf*)-deletion activates Hmgb1-Rage axis and granzyme A-mediated killing of pancreatic β cells resulting in neonatal lethality

**DOI:** 10.1371/journal.pone.0286109

**Published:** 2023-08-25

**Authors:** Gurvinder Kaur, Rebecca A. Helmer, Dalia Martinez-Marin, Souad R. Sennoune, Rachel L. Washburn, Raul Martinez-Zaguilan, Jannette M. Dufour, Beverly S. Chilton

**Affiliations:** 1 Department of Medical Education, Texas Tech University Health Sciences Center, Lubbock, Texas, United States of America; 2 Department of Cell Biology and Biochemistry, Texas Tech University Health Sciences Center, Lubbock, Texas, United States of America; 3 Department of Immunology and Molecular Microbiology, Texas Tech University-Health Sciences Center, Lubbock, Texas, United States of America; 4 Department of Cell Physiology and Molecular Biophysics, Texas Tech University Health Sciences Center, Lubbock, Texas, United States of America; University of Gondar, ETHIOPIA

## Abstract

Epigenetic mechanisms are integral to pancreatic β cell function. Promoter hypermethylation of the helicase like-transcription factor (*HLTF*) gene—a component of the cellular DNA damage response that contributes to genome stability—has been implicated in age-associated changes in β cells. To study HLTF, we generated global and β cell-specific (β) *Hltf* knockout (KO) immune competent (IC) and immune deficient (ID) *Rag2-/IL2-* mice. IC global and β *Hltf* KO mice were neonatal lethal whereas ID global and β *Hltf* KO newborn mice had normal survival. This focused our investigation on the effects of Rag2 interruption with common gamma chain interruption on β cell function/survival. Three-way transcriptomic (RNAseq) analyses of whole pancreata from IC and ID newborn β *Hltf* KO and wild type (*Hltf* +/+) controls combined with spatially resolved transcriptomic analysis of formalin fixed paraffin embedded tissue, immunohistochemistry and laser scanning confocal microscopy showed DNA damage caused by β *Hltf* KO in IC mice upregulated the Hmgb1-Rage axis and a gene signature for innate immune cells. Perforin-delivered granzyme A (GzmA) activation of DNase, Nme1, showed damaged nuclear single-stranded DNA (γH2AX immunostaining). This caspase-independent method of cell death was supported by transcriptional downregulation of *Serpinc1* gene that encodes a serine protease inhibitor of GzmA. Increased transcriptional availability of complement receptors *C3ar1* and *C5ar1* likely invited crosstalk with Hmgb1 to amplify inflammation. This study explores the complex dialog between β cells and immune cells during development. It has implications for the initiation of type I diabetes *in utero* when altered gene expression that compromises genome stability invokes a localized inflammatory response.

## Introduction

A balance between genome stability and genome diversification is required for survival of the species. Genome stability is the ability to preserve and faithfully transmit genetic material from cell to cell which includes Helicase-like transcription factor (HLTF) in error-free replication of DNA and the repair of damaged DNA [1]. Genomic instability is a hallmark of most cancers [2], and epigenetically silenced HLTF occurs frequently in colorectal cancer [3]. Initially, HLTF was identified as an E3 ubiquitin ligase that polyubiquitinylated PCNA and stimulated error-free post replication repair [4]. Subsequently, HLTF was shown to mediate replication stress via its fork reversal activity [5] and removal of damage-containing oligonucleotides to facilitate nucleotide excision repair [6].

DNA damage resulting from replication stress can result in increased DNA damage repair. Conversely, unrepaired DNA damage promotes cell death by apoptosis. DNA fragmentation in the later phase of apoptosis [7] can lead to the aberrant release of DNA fragments in the cytoplasm and trigger innate immune responses [8]. High mobility group box 1 (Hmgb1), a chromatin binding non-histone nuclear protein, the second most abundant protein in the nucleus, is passively released into the extracellular microenvironment during apoptosis [9]. Once outside the cell, Hmgb1, a damage associated molecular pattern molecule (DAMP), engages the pattern recognition receptor for advanced glycation end products (RAGE), to form a proinflammatory axis and alerts the innate immune system to excessive deregulated cell death [10]. Excessive activation of the innate immune signaling can lead to the development of autoimmunity [11].

In this regard, pancreatic β cells have been implicated in their own demise [12]. Type 1 diabetes (T1D) has long been described as an autoimmune disease in which the β cells are mistakenly destroyed by immune cells. However, it is possible that an epigenetic event in β cells could alter their dialog with islet-resident immune cells. Macrophages that initially function in a mediator role removing catabolic products could now respond to DAMPs leading to β cell destruction. Two lines of evidence support this concept. The first is the long-standing fact that genetic changes only account for ~50% of T1D [13]; and the second is compromised immune homeostasis in the islet microenvironment implicates immune surveillance in the pathology of T1D [14]. Therefore, we examined the role of Hltf, a known epigenetic target, in β cell death/survival using various KO models including β cell-specific KO mice and found a role of Hltf in preventing DNA damage thereby promoting β cell survival.

## Materials and methods

### Reagents and kits

Infrared warming pads were from Kent Scientific (Torrington, CT). OneTouch Ultra Mini and OneTouch Ultra Mini Blue test strips for the measurement of blood sugar were from LifeScan (Malpitas, CA). MiniCollect® red top capillary blood collection system (Z serum Clot Activator 450470, Greiner Bio-One) in combination with the MiniCollect® capillary tubes (450431) were from Summus Henry Schein (Melville, NY). Genomic DNA from tail biopsies was isolated with the DNeasy® Blood & Tissue Kit (69506) purchased from Qiagen (Valencia, CA). RNeasy® FFPE Kit (73504) and Deparaffinization Solution (19093) were also purchased from Qiagen. Invitrogen RNAlater stabilization solution (7020), and SequalPrep™ Long PCR Kit with dNTPs (A10498) were from ThermoFisher Scientific (Grand Island, NY). Midland Certified Reagent Company (Midland, TX) synthesized the PCR primers. MetaPhor™ Agarose (50180) was from LONZA (Rockland, ME). Promega (Madison, WI) was the source of the Lambda DNA/EcoRI + HindIII agarose gel markers (G173A). ALPCO was the source of mouse ultrasensitive insulin ELISA kits (80-INSMSU-E01). Vectastain® ABC-HRP Kit, Peroxidase (Guinea Pig IgG; PK-4007), Vectastain® ABC-HRP Kit, Peroxidase (Standard; PK-4000), and Hematoxylin QS (H-3404) were purchased from Vector Laboratories (Burlingame, CA). Diaminobenzidine (HK542-XAKE) was from BioGenex (Fermont, CA). Invitrogen by Thermo Fishcer Scientific was the source of ProLong™ Gold antifade reagent with DAPI (P36935). The DeadEnd^TM^ Fluorometric TUNEL assay (Apoptosis Detection System, G3250) was from Promega Corporation (Madison, WI). 10X Genomics (Pleasanton, CA) was the source of all Visium reagent kits for whole transcriptome profiling of intact formalin fixed paraffin embedded (FFPE) tissue sections including test slide (PN-1000347), slide kit (PN-1000188), reagent kit (PN-1000361), mouse transcriptome probe kit (PN-1000365), accessory kit (PN-1000194) and dual index kit TS Set A (PN-1000251). KAPA SYBR FAST qPCR master mix (KK4600) was purchased from Roche Diagnostic Corporation (Indianapolis, IN). SPRIselect (B23317) was purchased from Beckman Coulter Life Sciences (Indianapolis, IN). IgG-free, protease-free bovine serum albumin (BSA, 001-000-162) was purchased from Jackson ImmunoResearch (West Grove, PA).

Primary and secondary antibodies used with FFPE tissue sections in immunohistochemistry (IHC-P) and immunofluorescence (IF) are listed in Table 1. All protocols are accessible in protocols.io

DOI: dx.doi.org/10.17504/protocols.io.kxygx9yqdg8j/v1.

**Table 1:** Source, application and concentration of antibodies.

Primary antibodies	Secondary antibodies
Dako/Agilent Technologies (Santa Clara, CA) R.T.U. (ready-to-use) polyclonal guinea pig anti-swine insulin #IR002, IHC-P/IF 1:1000	IHC-P: Vector Laboratories RTU Biotinylated Goat Anti-rabbit IgG (H+L) (BP-9100-50) Invitrogen Alexa Fluor 647 goat anti-guinea pig (A21450) from ThermoFisher Scientific (1:200) Molecular Probes Alexa Fluor 488 Phalloidin (A12379) from ThermoFisher Scientific (1:500) in PBS-BSA 1%
Abcam (Cambridge, MA) rabbit monoclonal anti-γH2AX (phospho S139) ab81299 IHC-P 1:50 Bioss Antibodies Inc. (Woburn, MA) Rabbit polyclonal anti-Granzyme A (BS-2578R) from ThermoFisher IHC-P 1:200 Proteintech Rabbit polyclonal anti-NME1 (11086-2-AP) from ThermoFisher Scientific IHC-P 1:20	IHC-P: Vector Laboratories RTU Biotinylated Goat Anti-rabbit IgG (H+L) (BP-9100-50)

### *Hltf*-deleted mouse models and controls

IC global *Hltf* KO mice were developed in collaboration with genOway (Lyon, France) as previously described [15, 16]. Mice with either the *Hltf*-deletion or floxed *Hltf*-gene were bred to the recombinase activating gene 2 (*Rag2*)/common gamma (*IL2rg*) double knockout mice [17], thereby generating ID *Hltf* KO or *Hltf*-*fl/fl* mice. Mice with a floxed Hltf-gene that were either IC or ID were bred to Rip-Cre^TG^ mice (The Jackson Laboratory, Stock No. 003573) thereby deleting Hltf selectively in β cells (β *Hltf* KO)

All mice are in sentinel-monitored, rodent housing in the Laboratory Animal Resource Center (LARC) at Texas Tech University Health Sciences Center (TTUHSC). Additionally, mice on the *Rag2*)/*IL2rg* double knockout background are in bioBubble™-husbandry conditions in the LARC. Pathogen free mice were able to access food and water *ad libitum*. All studies were in accord with the NIH Guidelines for the Care and Use of Laboratory Animals, as reviewed and approved by the Animal Care and Use Committee at TTUHSC [NIH Assurance of Compliance A3056-01; USDA Certification 74-R-0050, Customer 1481, S1 Checklist]. TTUHSC’s IACUC specifically approved this study. Pain and suffering were always minimal. We previously reported the surgical removal of unborn pups and their placentae [17] from term pregnant females (n = 2). Each female received an IP injection of a Ketamine/Xylazine cocktail at 100 microliters per 20 g body weight. The cocktail consisted of 87.5 mg/kg Ketamine and 12.5 mg/kg Xylazine. Results from studies with the placentae were already published [17], whereas histology of pancreata from pups is reported here. Previously pregnant females were euthanized by drug overdose followed by cervical dislocation.

### Genotyping

PCR screening reaction to authenticated the ID *Hltf* KO genotype is as previously described [15–18] except we used SequalPrep reagents. PCR screening reactions were used to detect amplicons unique to the *Hltf floxed* allele (329-bp wildtype, 329/424-bp heterozygous, 424-bp floxed), and the *rIPCre* transgene (550-bp). Each 50 μl PCR reaction consisted of genomic DNA (60 ng), primer pairs (15 pmol each, Table 2), SequalPrep Long Reaction Buffer with nucleotides (5 μl of 10X), SequalPrep Long Enhancer B (2.5 μl), DMSO (1 μl), SequalPrep Long Polymerase (1 μl = 5 U). Reaction conditions for the *Hltf* floxed allele were as follows: 120 sec at 94°C, followed by 35 cycles of 94°C for 30 sec, 65°C for 30 sec, and 68°C for 120 sec, and a final extension for 480 sec at 68°C. Reaction conditions for the rIPCre transgene were as follows: 360 sec at 94°C followed by 40 cycles of 94°C for 60 sec, 60°C for 30 sec, and 72°C for 30 sec, and a final extension for 420 sec at 72°C. At the conclusion of each reaction, samples were cooled rapidly to 4°C, and amplicons were resolved/visualized by MetaPhor™ agarose (2%) gel electrophoresis with ethidium bromide (0.05 μg/ml).

**Table 2:** PCR primers for genotyping and gender authentication.

**Primers**	
*Hltf*-*floxed* forward	5’-ACC TCA ATT GAC ATC TTA ATC GGT CG-3’
*Hltf*-*floxed* reverse	5’-CTG CCA AGA TAC TCC AAA TCT GTT CAC TAC-3’
*rIPCre* forward	5’-CTC TGG CCA TCT GCT GAT CC-3’
*Cre 102* reverse	5’-CGC CGC ATA ACC AGT GAA AC-3’
*Myog* forward	5’-TTA CGT CCA TCG TGG ACA GC-3’
*Myog* reverse	5’-TGG GCT GGG TGT TAG TCT TA-3’
*Sry* forward	5’-TCA TGA GAC TGC CAA CCA CAG-3’
*Sry* reverse	5’-CAT GAC CAC CAC CAC CAC CA-3’

### Serum collection from newborn mice

Postprandial newborn mice are unable to thermoregulate, and were placed on infrared warming pads (37°C) to avoid the negative effects of hypothermia on blood glucose prior to decapitation with surgical scissors. Blood glucose in trunk blood was measured immediately in all members of each litter with the exception of pups that were already dead. Low (≤15 mg/dL) blood sugar in global and β *Hltf* KO mice affected anywhere from one pup in the litter to the entire litter. We used the MiniCollect® capillary blood collection system to collect trunk blood. Serum was removed from clotted blood after centrifugation. Serum samples (5–30 μl) were stored frozen (-20°C) until use in an ultrasensitive insulin test. Tails from pups were used for genotyping and gender authentication.

### Mouse ultrasensitive insulin ELISA

The Mouse Ultrasensitive Insulin ELISA (Alpco) quantified the concentration of insulin protein from mouse I and mouse II proinsulin genes according to the manufacturer’s instructions. There was no cross reactivity with mouse C-peptide 1 or 2, or mouse IGF 1 or 2. Because 25 μl of serum was required for hypoinsulinemic samples, it was necessary to pool serum samples in a gender specific manner as shown in Table 3.

**Table 3:** Number (N) of samples in blood sugar and serum insulin calculations.

Genotype	N animals = N blood glucose values	N = Insulin from pooled serum
IC *Hltf +/+* (control)	231	69
IC Global *Hltf* KO	209	26
IC β *Hltf* KO	182	22
IC *Hltf fl/fl* (control)	193	43
IC *rIPCre Hltf +/+ (control)*	191	43
ID *Hltf+/+* (control)	87	27
ID *Global Hltf* KO	183	27
ID β *Hltf* KO	303	109

### Analysis of pancreatic tissue

Abdominal segments of IC *Hltf* +/+ (control) and global *Hltf* KO E18.5 mice (N = 8/group) were formalin fixed overnight at 4°C, paraffin embedded, and serially sectioned (4 μm). Tissue sections were either stained with Hematoxylin and Eosin (H&E), or immunostained for insulin. Diaminobenzidine was the chromogen.

Abdominal segments of newborn IC pups were infused with formalin-based fixative (n = 12 each) for global *Hltf*-KO with low (≤15 mg/dL) blood sugar, *Hltf* +/+ control, and β *Hltf* KO with low blood sugar. In companion experiments, abdominal segments of ID new born pups of the same three genotypes were infused with formalin-based fixative. All tissues were fixed overnight at 4°C, paraffin embedded, and serially sectioned (4 μm).

For tissue insulin quantification, tissue sections were subjected to heat-induced epitope retrieval (HIER) with citrate buffer pH 6.0, then immunolabeled (Table 1). Slides were then incubated with 4′, 6-diamidino-2-phenylindole, dihydrochloride (DAPI; 1 μg/mL) to detect cell nuclei. Images at 20x magnification were merged, and quantified with Image J software.

Immunocytochemistry (Table 1) for laser scanning confocal microscopy was performed with serial sections from the above described groups of newborn pups with HIER, aldehyde quench (50 mM NH_4_Cl in PBS), and ProLong Gold DAPI.

### TUNEL assay

Apoptosis was determined using the DeadEnd^TM^ Fluorometric TUNEL assay with slides from the samples used for quantification of insulin expression (above) according to the manufacturer’s instructions. Negative controls included sections incubated without the TdT enzyme, and were devoid of a positive reaction. For quantification, the area of TUNEL positive cells was determined using particle analysis (internal function of Image J) in pixels^2^ for each image. Contrast enhancement expanded the dynamic range of images, and color threshold was set at a constant value, which only selected the positive staining areas. To control for tissue size, the total area of TUNEL positive cells was normalized to the total tissue area.

### Statistical analysis

All values are expressed as the mean ± standard error of the mean (SEM) of *n* independent experiments. With the exception of RNA-seq and spatial transcriptomics, all data analyses were conducted with GraphPad Prism version 9.1.1 software. For multiple comparisons, we performed a one-way analysis of variance (ANOVA) with an appropriate *post hoc* test as described for each experiment, *p*<0.05 was significant.

### Pancreatic transcriptome

Because pancreata are ribonuclease-rich [19], trunks of decapitated newborn mice were perfused *in situ* with RNAlater by insertion of a 20g-1-inch needle attached to a 5 ml syringe into the abdomen [20] via the crural (posterior) attachment of the diaphragm [21]. RNA stabilization occurred concomitant with the initial stretching of the pancreas. Pancreata were stored in RNA later at -70C until total RNA was isolated. RNA integrity and purity were assessed (Agilent Bioanalyzer) for 10 samples, i.e. 3 from β *Hltf* KO IC mice with low blood sugar, 3 from *Hltf +/+* controls, and 4 from β *Hltf* KO ID mice. cDNA was generated from Ribo-Zero Plus rRNA-depleted samples and subjected to Illumina library preparation. Libraries were sequenced utilizing Illumina sequencing technology. Paired-end 100 nucleotide reads were aligned to reference mouse genome C57BL/6J (GRCm38/mm10) and analyzed using the platform provided by DNAnexus, Inc. (Mountain View, CA) to generate three-way transcriptomic (RNAseq) analyses of whole pancreata from β *Hltf* KO IC and ID newborn mice, and wild type (*Hltf* +/+) controls. The analysis included alternative splicing analysis in control (*Hltf +/+*) pancreata. The power in detecting alternative splicing was dramatically increased by paired-end sequencing relative to single-end sequencing. FPKM (fragments per kilobase of transcript per million mapped reads) were mapped against mm10 with Tophat (V1.3.3) to obtain.bam mapping files that were input into Cufflinks for transcript assembly. Cuffdiff (V 1.3.0), part of the Cufflinks package, used the alignment reads for rigorous statistical comparison of the three genotypes. The depth of sequencing (Table 4) was a minimum of 20 million sequencing reads per sample [90% Power, 5% significance level: 91+/- 4% of all annotated genes are sequenced at a frequency of 0.1 times/10^3^ bases X 3 x 10^9^ bases/sequencing read x 3 samples = 9 x10^4^ reads/gene]. All RNA-seq data were deposited in NCBI’s Gene Expression Omnibus and are accessible through GEO Series accession number GSE137060. (https://www.ncbi.nlm.nih.gov/geo/query/acc.cgi?acc=GSE137060)). Data were imported into iPathwayGuide (Advaita Corporation) a next-generation pathway analysis tool. Standard enrichment parameters (log2 fold change, log2 FC = 0.6, p<0.05) were used.

**Table 4:** Sample quality control and RNAseq outcomes.

Sample ID	OD260/280	RIN^a^	Total Bases	Total Reads
1 IC β *Hltf* KO	2.08	6.3	2,217,676,244	20,921,474
2 IC β *Hltf* KO	2.08	6.2	2,669,141,056	25,180,576
3 IC β *Hltf* KO	2.09	5.9	2,742,187,988	25,869,698
4 *Hltf* +/+ (control)	2.06	6.2	3,035,583,268	28,637,578
5 *Hltf* +/+ (control)	2.11	7.1	2,934,994,992	27,688,632
6 *Hltf* +/+ (control)	2.06	6.4	2,794,437,508	26,362,618
7 ID β *Hltf* KO	2.11	6.4	12.654,850,960	83,806,960
8 ID β *Hltf* KO	2.11	6.5	10,603,328,720	70,220,720
9 ID β *Hltf* KO	2.09	8.6	13,039,568,156	86,354,756
10 ID β *Hltf* KO	2.11	8.8	12,657,228,002	83,822,702

^a^An RNA integrity number (RIN) from an Agilent Bioanalyzer

### Spatial transcriptomics

#### Work flow

Five basic steps were necessary to implement spatial transcriptomics technology. Step 1, placement of FFPE tissue (abdominal segments) on capture areas of a Visium gene expression (GEX) slide. Step 2, H&E staining followed by brightfield microscopic imaging with ZEISS Axioscan 7 high-performance slide scanner (White Plains, NY). Step 3, permeabilization of tissue and construction of barcoded libraries with a final sample index PCR all according to the manufacturer’s instructions. Step 4, NGS short-read sequencing (Illumina NovaSeq) of barcoded libraries by Genewiz (Azenta US, Inc, South Plainfield, NJ). Step 5, data analysis of tissue images and sequencing files in FASTQ format with Space Ranger run on Ubuntu 22.04 LTS–Thelio Mira-b3 by System76, Inc. (Denver, CO). The space ranger aggr pipeline was used to aggregate data from replicate samples and from samples from the different biological conditions (IC, ID). Loupe browser visualization software was accessed in a desktop application via Windows (Dell Optiplex 990).

#### FFPE sections

Abdominal tissue sections (5 μm) from IC and ID β *Hltf* KO newborn mice were processed with the RNeasy FFPE kit for DV200 analysis. Replicate sections from IC and ID β *Hltf* KO newborn mice were placed within fiducial frames of capture areas A,B and C,D respectively, on Visium GEX slide V11D13-089-A1. 10X Genomics best practices guide helped to maintain tissue adhesion and RNA integrity before and after sectioning.

#### GEX slide

Four capture areas (6.5 x 6.5 mm each) inside fiducial frames measure 8 x 8 mm. Each capture area contains 5,000 gene expression spots (55 μm in diameter) spaced with a distance of 100 μm between the centers of each spot and captures gene expression data for 1–10 cells. Visium for FFPE uses RNA-templated ligation (RTL) probes targeting the whole transcriptome. The assay does not capture transcripts directly, but captures probes via a capture sequence, e.g. poly-A for Visium for FFPE probes. Each gene expression spot has primers with a unique spatial barcode Probes are designed against the entire mouse genome, each with primers that include Illumina TruSeq Read 1 (partial read 1 sequencing primer), 16 nt spatial barcode (all primers in a specific spot share the same spatial barcode), 12 nt unique molecular identifier (UMI), and 30 nt poly(dT) sequence (captures ligation product). Spatially barcoded, ligated products were released from the slide, and harvested for qPCR with KAPA SYBR Fast qPCR master mix. The threshold for determining the Cq value for each sample was set along the exponential phase of the amplification plot at ~25% of the peak fluorescence value with QuantStudio 12 K Flex real-time PCR system (ThermoFisher Scientific). Sample index sets were selected to distinguish each of the 4 samples in a multiplexed sequencing run. Samples were amplified using Ilumina-compatible indexing primers, cleaned up with SPRIselect reagent, and bi-directionally sequenced.

#### Mouse probe set

Visium Mouse Transcriptome Probe Set v1.0 contains 20,551 gene ids targeted by 20,873 probes. Gene ids (1,086, 5.3%) targeted by 1,110 probes were excluded by default due to predicted off-target activity to a different gene. As a result, 19,465 gene_ids (targeted by 19,763 probes) were present in the final filtered output. During data analysis, read 2 sequences were mapped against the reference mouse genome C57BL/6J (GRCm38/mm10) and read 1 sequences were used for UMI filtering to obtain spatial information.

#### Sequencing

Illumina NovaSeq at GenWiz (Azenta Life Sciences, South Plainfield, NJ). Unique dual indexing—unique identifiers on both ends of the sample—allows for an increase in the number of samples sequenced per run and reduces per-sample cost compared to other indexing strategies. Sequencing depth was a minimum of 50k read pairs per spot covered with tissue. This was calculated by estimating the percent of capture area covered by the tissue section based upon the H&E brightfield image. Actual values are provided in Table 5.

**Table 5:** Statistics for spatial transcriptomics outcome for Visium_FFPE_Mouse_Pancreas in ID β *Hltf*-KO (Sample ID, A and B) and IC β *Hltf*-KO (Sample ID, C and D).

ID	DV200	# spots under tissue	Mean reads/spot	Median genes/spot	# of reads^a^	Validated barcodes	Sequencing saturation	Genes Detected
A	55	2,312	342,205	1,404	791,177,066	98.1%	100.0%	18,382
B	55	1,932	490,660	1,224	947,955,384	98.0%	99.2%	17,949
C	65	2,833	147,348	3,192	417,437,493	98.0%	92.3%	18,463
D	65	3,110	237,777	3,514	739,485,162	98.3%	94.6%	18,887

^a^100% of UMIs valid for each sample

#### Bioinformatics analysis

Bioinformatic analysis utilized the Visium Spatial Gene Expression Software Suite that includes Space Ranger and Loupe Browser. Space Ranger has three pipelines for FFPE data analysis.

Spaceranger mkfastq demultiplexed the Illumina sequencer’s base call files (BCLs) for each flow cell directory into FASTQ files. Spaceranger count combined a brightfield microscope slide image and FASTQ files from spaceranger mkfastq and performed alignment, tissue detection, fiducial detection, barcode/UMI counting, and prepared a full resolution slide image for visualization in Loupe Browser. The pipeline used the Visium spatial barcodes to generate feature-spot matrices, determine clusters, and perform gene expression analyses. The pipeline uses a probe aligner algorithm for FFPE tissues. Spaceranger aggr used the output of multiple runs of spaceranger count from related samples and aggregated their input, normalizing those runs to the same sequencing depth, and then recomputed the feature-barcode matrices and the analysis on the combined data. The aggr pipeline combined data from multiple samples into an experiment-wide feature-barcode matrix and analysis. Loupe Browser was used to interrogate significant genes, characterize and refine gene clusters, and perform differential expression analyses.

## Results

### Perinatal lethal phenotype

**Fig 1:**
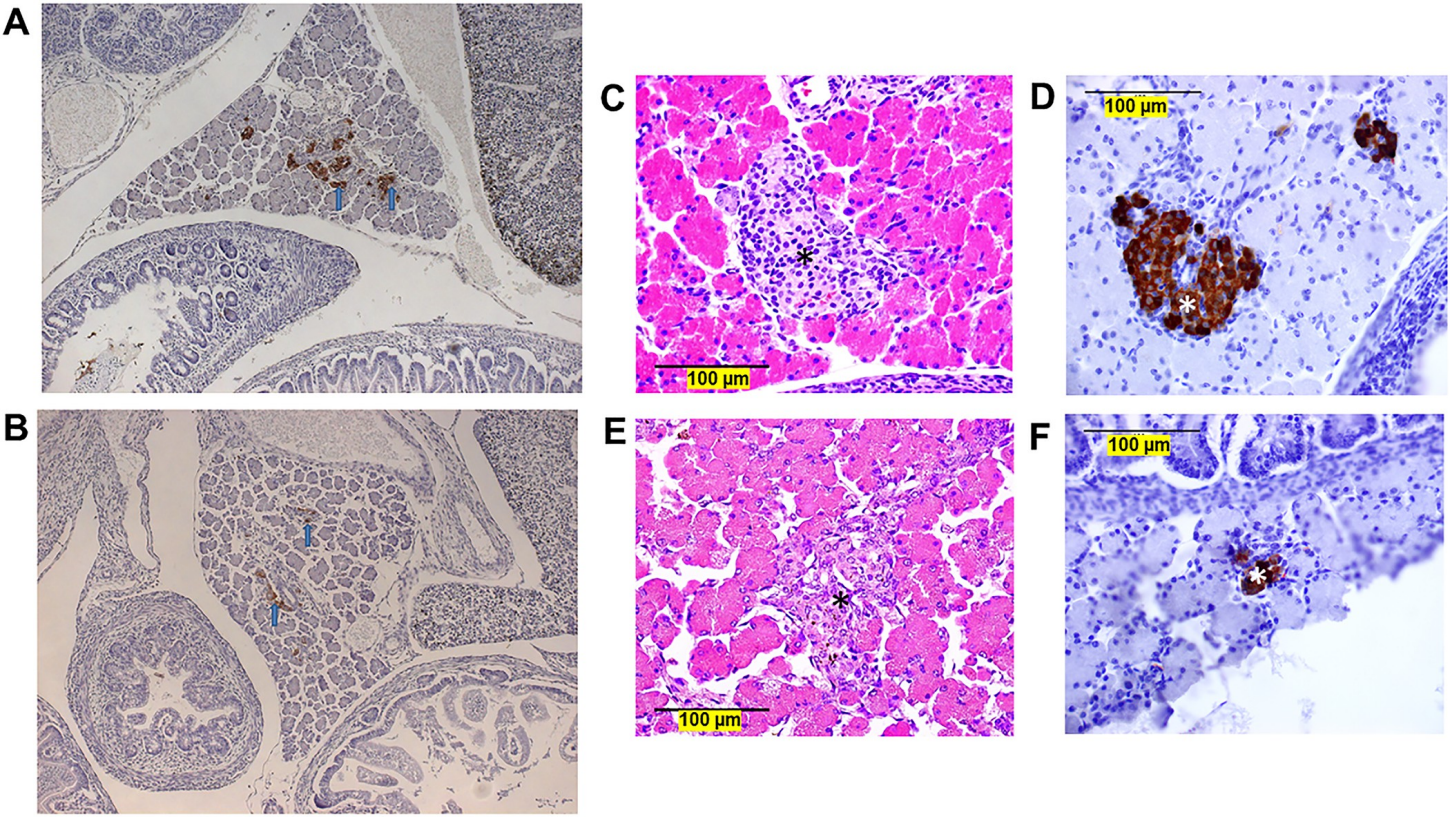
Pancreata from E18.5 mice. Sections from IC control (A) and global *Hltf* KO (B) embryonic mice were immunostained for insulin (blue arrows), counter stained with hematoxylin, and photographed at 10X magnification. Pancreata from IC control (C and D) and global *Hltf* KO (E and F) fetal mice were stained with either H&E for normal histology (C and E), or immunostained for insulin and counterstained with hematoxylin (D and F), 40X magnification. Control and global *Hltf* KO pancreata contain both acini and islets with β cells (asterisk,*). Size marker = 100 μm.

Phenotypic analysis of global *Hltf* KO mice was challenging because the gene deletion caused perinatal death. Survivors have contributed to our understanding of *Hltf* gene function in cancer [22, 23]; however, until now, the exact mechanism responsible for perinatal death has remained obscure. Although a single gene deletion can alter more than one physiological system, studies on brain [15], heart [16] and placenta [17] only served to eliminate *Hltf*-deletion from these organs as directly responsible for perinatal lethality. In contrast, morphological evaluation of pancreata on mouse embryonic (E) day 18.5, indicated the mechanism underlying the complex phenotype was established *in utero*. Microscopic observation (Fig 1) shows typical pancreatic organization with normal acinar and ductal tissue and insulin-positive islets containing β cells in *Hltf* +/+ (control) and IC global *Hltf* KO mice. However, upon closer inspection, the islets in control tissue are more robust compared to the islets in IC global *Hltf* KO mice that appear smaller with distorted histomorphology (Fig 1C–1F). Reduced insulin expression was evident in pancreata from IC global *Hltf* KO at both low (Fig 1B) and high (Fig 1F) magnifications compared with controls (Fig 1A and 1D).

### Pancreata in newborn mice

**Fig 2:**
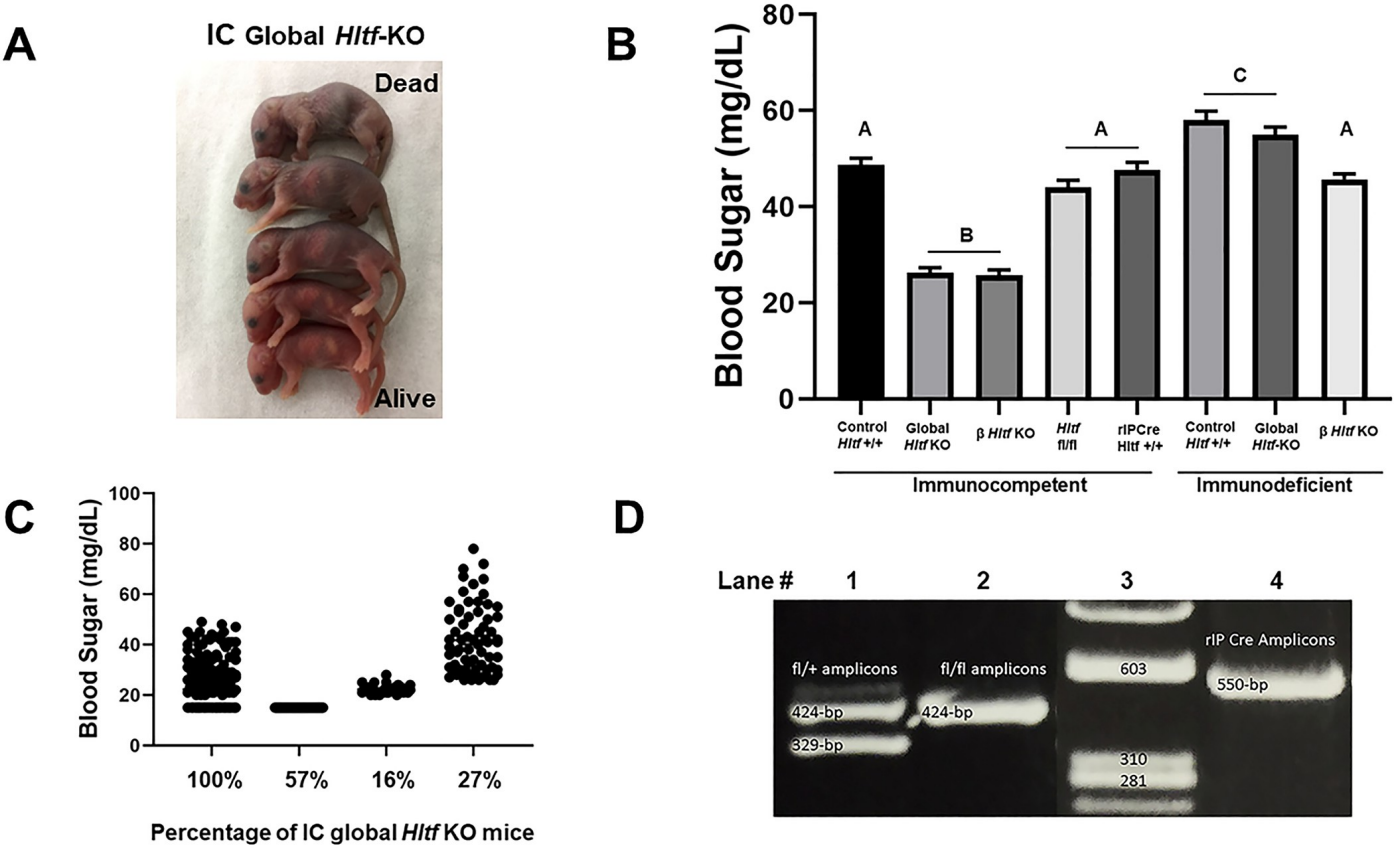
Composite phenotype. (A) Appearance of postprandial newborn IC *Hltf* KO mice. Note milk-filled stomach (milk spot) in each pup, which indicates they were born alive and capable of suckling. (B) ANOVA analysis (p<0.0001) and Tukey’s multiple comparisons test of blood sugar shows reduced blood sugar levels are identical (p = 0.9999) for IC global and β *Hltf* KO mice. Findings confirm negligible effects of the *rIPCre* transgene on blood sugar levels in newborn mice. Blood sugar levels for ID control and global *Hltf* KO pancreata do not differ (p = 0.9261) from each other compared to β *Hltf* KO pancreata that differ (p<0.0001) from the other two values but are comparable (p = 0.6520) to IC Hltf +/+ (control) mice. Values are mean ± SEM, and values with the same letter designation are not significantly different (p>0.05). (C) Data from IC global *Hltf* KO mice show the percentage of mice with low blood sugar is nearly identical to the rate of neonatal lethality. (D) PCR validation of genotypes with amplicons of the expected sizes shown for *fl/+* genotype (Lane 1), *fl/fl* genotype (Lane 2), ϕX174 DNA/Hae III molecular size markers (Lane 3) and *rIPCre* transgene (Lane 4).

Newborn IC global *Hltf* KO mice and their littermate controls are pink in color and display a sucking reflex immediately after birth. Despite the presence of milk in their stomachs, 75% of newborn IC global *Hltf* KO mice lose their surface righting reflex, and display central cyanosis (Fig 2A), consistent with hypoglycemia (Fig 2B). Reevaluation of blood sugar data from IC global *Hltf* KO mice (Fig 2C) shows the percentage of mice with low or marginal (20–25 mg/dL) blood sugar levels comprised 73% of the population of IC global *Hltf* KO mice. With the minimum threshold set at 26 mg/dL, 27% of the total population of newborns achieved normal blood sugar commensurate with the survival statistics. Importantly, of the 57% of newborn mice with low blood sugar (Fig 2C), males were affected more frequently (3:2 ratio) than females. This finding is consistent with the unexplained fact that diabetes is more frequent in men than women. *rIPCre* transgenic mice were used to generate β *Hltf* KO mice in order to eliminate potential global *Hltf*-deletion effects—on gut hormones, glucagon secretion, nutrient-sensing neurons in glucose homeostasis, and glucose uptake by liver, adipose and muscle—that would alter glucose metabolism. Fig 2D shows PCR validation of the genotype. Because *rIPCre* mice alone display glucose intolerance as early as 6 weeks of age [24, 25], it was imperative to include all the appropriate controls to show *Hltf* fl/fl as well as *rIPCre Hltf +/+* mice have normal blood sugar at birth compared with *Hltf* +/+ control pups (Fig 2B). IC global and β *Hltf* KO mice share the same dramatic reduction in circulating levels of blood glucose and the same perinatal lethal phenotype indicating it is solely attributable to alterations in islet β cells (Fig 2B).

**Fig 3:**
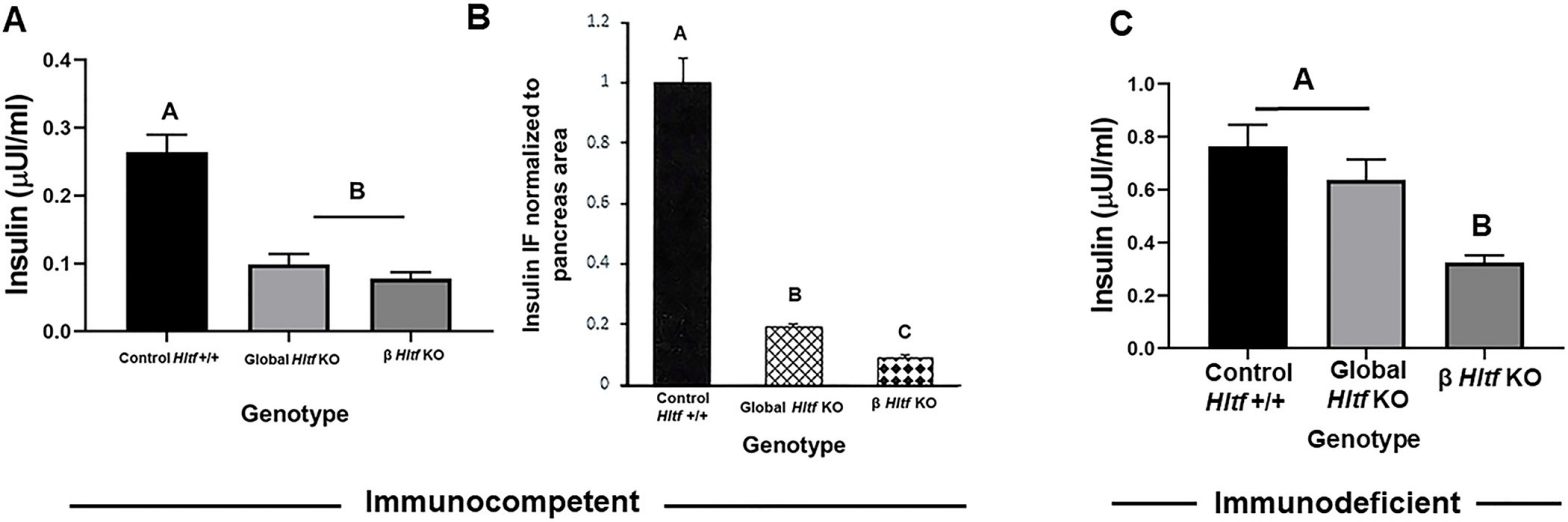
Comparison levels of insulin in serum and pancreata. (A) The ELISA assays had a dynamic range of 0.025–1.25 ng/ml and superior sensitivity of 0.019 ng/ml (25 μl samples). Assay precision was confirmed with intra-assay variation (CV % = 5) and inter-assay variation (CV % = 6) measurements. Standard curves (0.025, 0.09, 0.188, 0.5, and1.25ng/mL) with each ELISA plate consistently provided r = 0.999 values. ANOVA analysis (p<0.0001) and Tukey’s multiple comparison test (p<0.0001) indicate serum insulin levels are comparable for IC global and β *Hltf* KO mice. (B) ANOVA analysis and Fisher’s LSD test show the insulin content of pancreatic β cells is reduced in IC global (p<0.03978) and β *Hltf* KO mice (p<0.00001). These data are from quantification (Image J software) of insulin immunolabeling (immunofluorescence). (C) ANOVA analysis (p<0.0001) and Tukey’s multiple comparison test (p<0.0001) indicate serum insulin levels are comparable (p = 0.4846) for ID control and global *HLTF* KO but decreased (p = 0.0001) in ID β *HLTF* KO mice. As previously seen with serum glucose levels, serum insulin levels (p = 0.1271) do not differ from IC *Hltf* +/+ controls. Values in A, B and C are mean ± SEM. Values with the same letter designation are not significantly different (p>0.05).

Potential confounding effects of the innate immune system on pancreatic development/function were eliminated when the *Hltf*-deletion line was bred into the recombinase activating gene 2 (Rag2)/common gamma (IL2rg) double knockout background. Unexpectedly, the perinatal lethal phenotype was eliminated, i.e. newborn ID global and β *Hltf* KO mice were born euglycemic (Fig 2B) with survival rates equivalent to IC *Hltf* +/+ controls. Because IC global and β *Hltf* KO mice share the characteristic of low blood sugar levels compared to controls, we measured their non-fasting serum insulin levels and quantified insulin expression in their pancreata. As shown in Fig 3, serum insulin levels for IC global and β Hltf KO mice are reduced (Fig 3A and 3B). In comparison, newborn ID global and β *Hltf* KO mice were born normoinsulinemic (Fig 3C).

### Pancreatic transcriptome

To understand *Hltf*-deletion effects on β cell development and resultant changes in gene expression, we performed three-way transcriptomic (RNAseq) analyses of whole pancreata from IC β *Hltf* KO, ID β *Hltf* KO and IC control (*Hltf* +/+) newborn mice. The microenvironment (interstitial matrix, peri-islet basement membrane, and microvascular cells), as it was potentially altered by β cell-specific *Hltf* gene-deletion, was an integral component of the experiment. Using the iPathwayGuide analysis tool, RNA-seq data showed transcription of two genes most exclusively expressed in β cells—nonallelic insulin 2 (*Ins2*) and insulin 1 (*Ins1*) genes—as well as Neurogenin3 (Neurog3), sex-determining region Y (SRY)-related high mobility group (HMG) box (SOX) transcription factor 4 (Sox4), pancreatic polypeptide (Ppy)-lineage β cells and pancreatic and duodenal homeobox 1 (Pdx1) were transcriptionally downregulated in IC β *Hltf* KO mice. These data confirmed that *Hltf*-deletion in the presence of an intact immune system is associated with β cell loss during development. These findings are consistent with data showing reduced insulin hormone in whole tissue and in the systemic circulation. Additional findings were exclusionary. *Hltf*-deletion throughout embryonic development produced no altered molecular signature (*Ngn 3*, *Pou5f*, *and Mycl1*) suggesting the progenitor cell state was maintained. There was no effect of β cell-specific *Hltf*-deletion on transcription of glucagon (α cells) or somatostatin (δ cells) genes.

**Fig 4:**
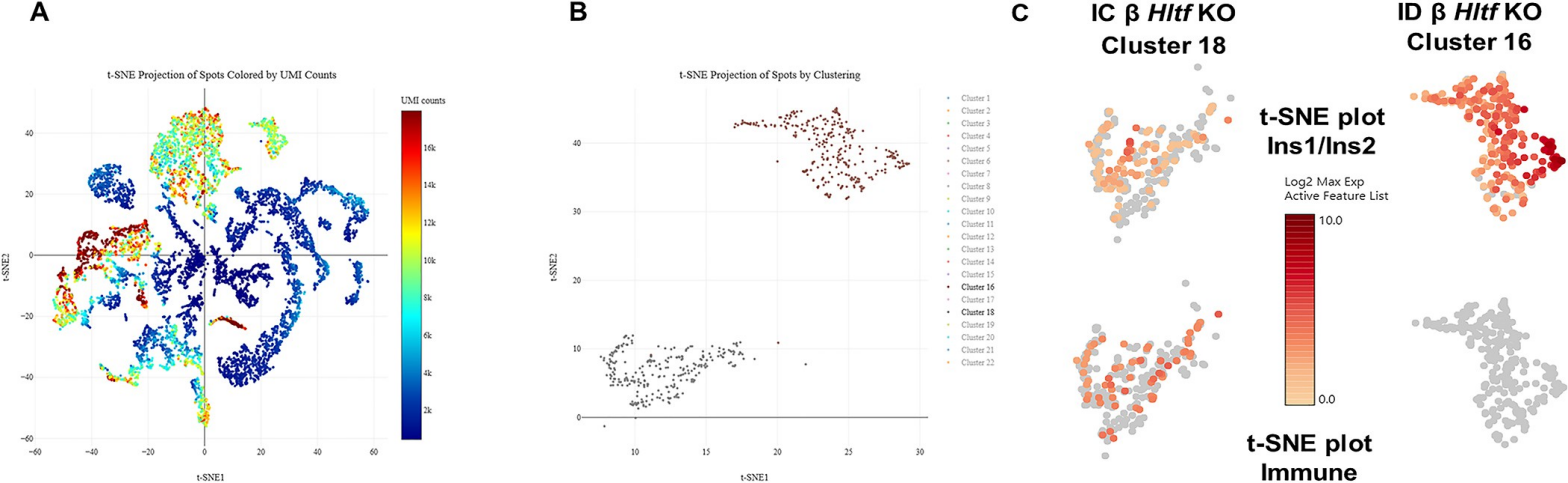
Exploration of the tissue architecture of IC and ID β *HLTF* KO pancreata. (A) Two-dimensional batch-corrected *t*-distributed stochastic neighbor embedding (*t*-SNE) visualization of the UMI counts from the entire IC vs ID dataset. (B) Pancreata clusters 16 and 18 are shown in *t*-SNE space. (C) Pancreatic genes insulin 1 (Ins1) and insulin 2 (Ins2) illustrated differential gene expression in IC vs ID β Hltf KO mice in *t*-SNE plots of clusters 16 and 18. The unique expression of immune cell markers in IC vs ID tissue is elaborated in *t*-SNE plots of these clusters. *Slamf6* and *Il2rb* were added to previously identified markers for NK cells (*GzmA*, *Klrb1b*). Five markers for B cells (*Pax5*, *Blk*, *Fcmr*, *Fcrla*, *Tnfrasf9*) and three markers for activation of innate immunity (*Bpifb1*, *Serpinb3a*, *Defb36*) were unique to cluster 18.

To accommodate the long expanses of interconnected islets located along large blood vessels in the neonatal pancreas [26] and to preserve neonatal intra-islet architecture, spatial transcriptomics—which incorporates unbiased total mRNA analysis in intact FFPE sections of abdominal segments from IC and ID β *Hltf* KO mice in a morphological context—globally distinguished 22 graph-based clusters (Fig 4A). Next, tissue transcriptomics and gene deconvolution were used to quantify differential gene expression in pancreata from IC (cluster 18) vs ID (cluster 16) samples (Fig 4B). Mapping insulin 1 and insulin 2 gene expression in islets from IC and ID β *Hltf* KO mice (Fig 4C) confirmed reduced insulin gene expression in IC β *Hltf* KO mice.

### Murine *Hltf* is alternatively spliced

**Fig 5:**
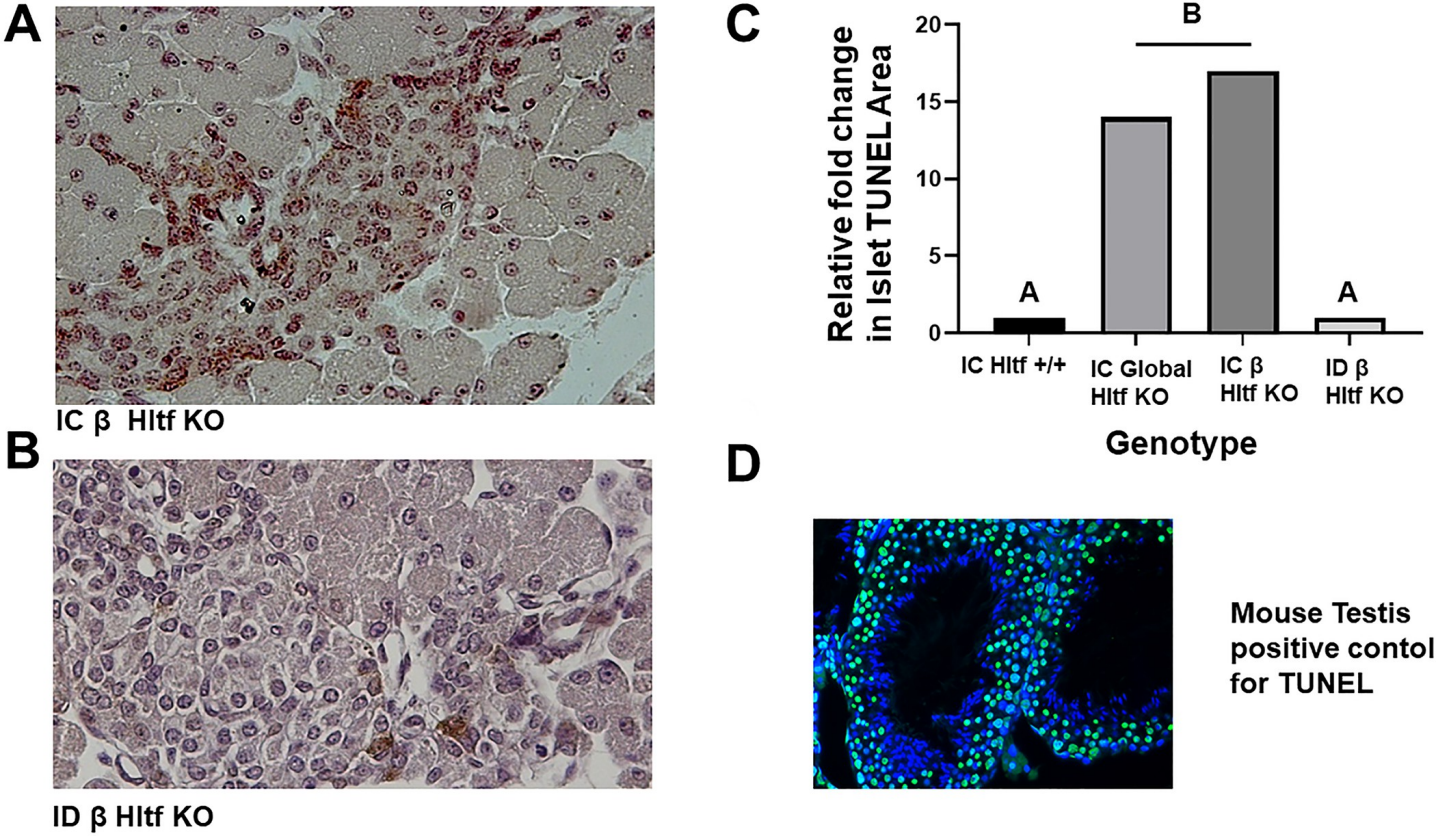
Differential γH2Ax pan-staining and TUNEL assay. Abundant γH2Ax in β cells from IC β *Hltf* KO mice (A) compared to minimal immunostaining in β cells from ID β *Hltf* KO mice (B). Two-types of γH2Ax pan-staining are evident. β cells from IC β *Hltf* KO mice have apoptotic rings and the β cells from ID β *Hltf* KO mice have limited pan-nuclear staining of the entire nucleus. Results from the terminal deoxynuceotidyl transferase dUTP nick-end labeling (TUNEL) assay (C), which detects β cell death-associated DNA fragmentation (3’-OH termini), indicates the amount of DNA damage is more than the targeted β cells can efficiently repair when the animals are IC. Cell-death in IC *Hltf +/+* controls and ID β *Hltf* KO mice was negligible. A positive mouse testis control (D) was included because apoptosis is an important component of normal spermatogenesis.

Post-transcriptional processing yields a full-length message isoform (4955-bp; exons 1–25) and a 3´-truncated isoform (3059-bp; exons 1–21 with exon 21 extended via an intron retention event) in mouse brain [15] and heart [16]. Term placenta exclusively expresses the short isoform [17]. The **full-length** mRNA encodes a full-length protein with a DNA repair domain. The short mRNA encodes a nearly identical truncated protein that lacks the DNA repair domain. *Hltf*-deletion of the **full-length** splice variant, confirmed by RNAseq, in pancreas precludes the protein’s DNA-damage response from benefiting β cells. DNA damage from double stranded breaks is quickly followed by phosphorylation of Ser-139 of the histone variant H2AX. Foci of the newly phosphorylated protein, known as γH2AX, is a hallmark of DNA damage associated with a germline *HLTF* mutation in familial myelodysplastic syndromes (MDS), disease-related depletion of *HLTF*, epigenetic silencing of *HLTF* in colorectal cancer and in experimental cell systems from which *HLTF* has been deleted. However, when *Hltf*-deletion effects were assayed by examining γH2AX expression the level of DNA damage was raised to a threshold that triggered apoptosis in β cells from IC β *Hltf* KO mice compared to ID β *Hltf* KO mice (Fig 5).

**Fig 6:**
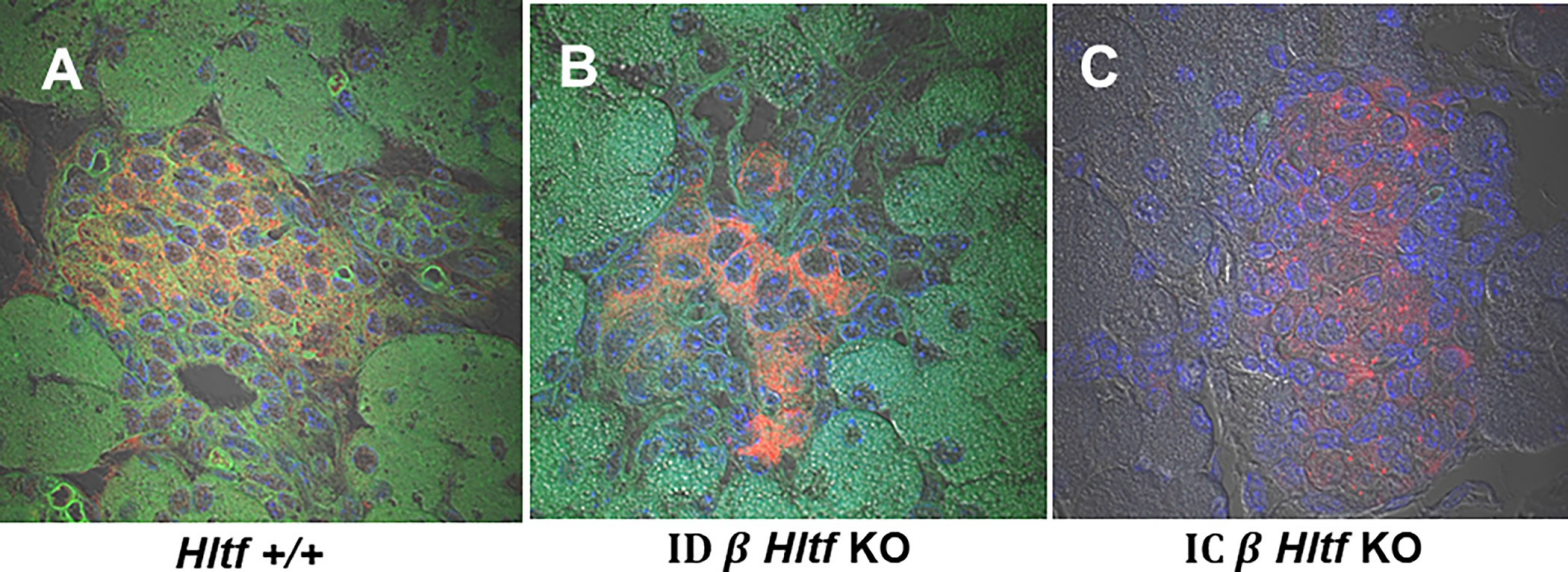
Nuclear morphology and DNA damage in response to β *Hltf* KO. The confocal image analysis was driven by the desire to visualize the integrity of nuclear DNA (DAPI, blue) in the context of the F-actin cytoskeleton (phalloidin, green) using insulin (red) as a definitive β cell identifier. DAPI binds the AT-rich regions of double-stranded DNA. Binding is accompanied by a 20-fold enrichment in fluorescence that is directly proportional to the amount of DNA as shown for Hltf +/+ (A) and ID *β* *Hltf* KO (B). In contrast, when the cell membrane is compromised by apoptosis, more DAPI enters the cells and stains a stronger blue color as shown for IC *β* *Hltf* KO (C). Chromosome condensation and DNA fragmentation enhances the visual identification of apoptotic cells stained with DAPI. Cell shrinkage occurs as a result of the serine/threonine kinase rearranging the cytoskeleton as visualized by staining for actin in merged immunofluorescence images. Colocalization (orange) of insulin (red) with the actin cytoskeleton (green) is shown for Hltf +/+ (A) and ID *β* *Hltf* KO (B). Actin is cleaved during late stage apoptosis thus actin immunostaining is negligible for IC *β* *Hltf* KO (C). For data presentation, the maximal projection confocal images obtained from a z stack (500 nm slice) using a 60x objective (oil) are shown. All images were obtained with transmitted light and excitation at 405 nm (DAPI), 488 (F-actin) and 647 nm (insulin).

Nuclear fragmentation (Fig 6), a morphological feature of apoptosis, has been classified as immunogenic cell death when associated with damage-associated molecular patterns (DAMPs) release. Comparative transcriptomic (RNAseq) analysis revealed significant upregulation of the Hmgb1-Rage axis in IC β *Hltf* KO cells resulting in the extracellular availability of Hmgb1. The log2 FC +0.839 (p = 0.04) in the high mobility group box 1 (*Hmgb1*) gene that encodes the prototypical danger molecule Hmgb1, coincided with the log2 FC +1.648 (p = 0.035) in the advanced glycosylation end product (*AGE*) receptor gene otherwise known as Rage. Extracellular Hmgb1 initiates an inflammatory response and activates dendritic cells. Comparative transcriptomics (RNAseq) confirmed gene signatures for dendritic cells, *Ctla-2a* (log2 FC +2.001, p = 0.004) and tissue resident macrophages—*Adgre1* (*Emr1*, F4/80; log2 FC +2.468, p = .001), *Ptprc* (Cd45; log2 FC +1.330, p = 0.034), *Itgam* (Cd11b; Log2 FC +1.900, p = 0.001)—indicative of endocrine macrophages—unaltered expression of the mannose receptor (*Mrc1*/Cd206/Mmr, p = 0.158) and increased *Csf1r* (log2 FC +1.264, p = 0.005)—concomitant with the expression of phagocytosis/efferocytosis-related genes *Pparg* (log2 FC +1.763; p = 0.018), *Stab2* (log2 FC +2.093, p = 0.005), *Cd59a* (log2 FC +2.013, p = 0.006), and *Arg1* (log2 FC +1.733, p = 0.008) in pancreata of IC β *Hltf* KO mice.

Most importantly, RNA-seq in combination with spatial transcriptomics identified 8 members of a13 natural killer (NK) cell-specific gene signature [27] encoding—three (3) lectin-like activating receptors *Klrb1c* (Nk1.1), Klrk1 (Nkg2D), *Ncr1* (Nkp46); one lectin-like inhibitory receptor Klrb1b; a cytotoxic phenotype characterized by transcription factors eomesoderminin (*Eomes*), perforin (*Prf1*) and granzyme A (*GzmA*); *IL-18* and its co-receptor (*Il18rap*)—plus expression of T-box transcription factor 21 (*Tbx21*) and the proinflammatory cytokine interferon (*Ifn*)-*γ*—in pancreata of IC β *Hltf* KO mice. Overlapping and additional immune cell transcriptome signatures were identified with an Interacting Multiple-Model (IMM) filter (ClueGo analysis) of spatial transcriptomics data (Fig 4).

**Fig 7:**
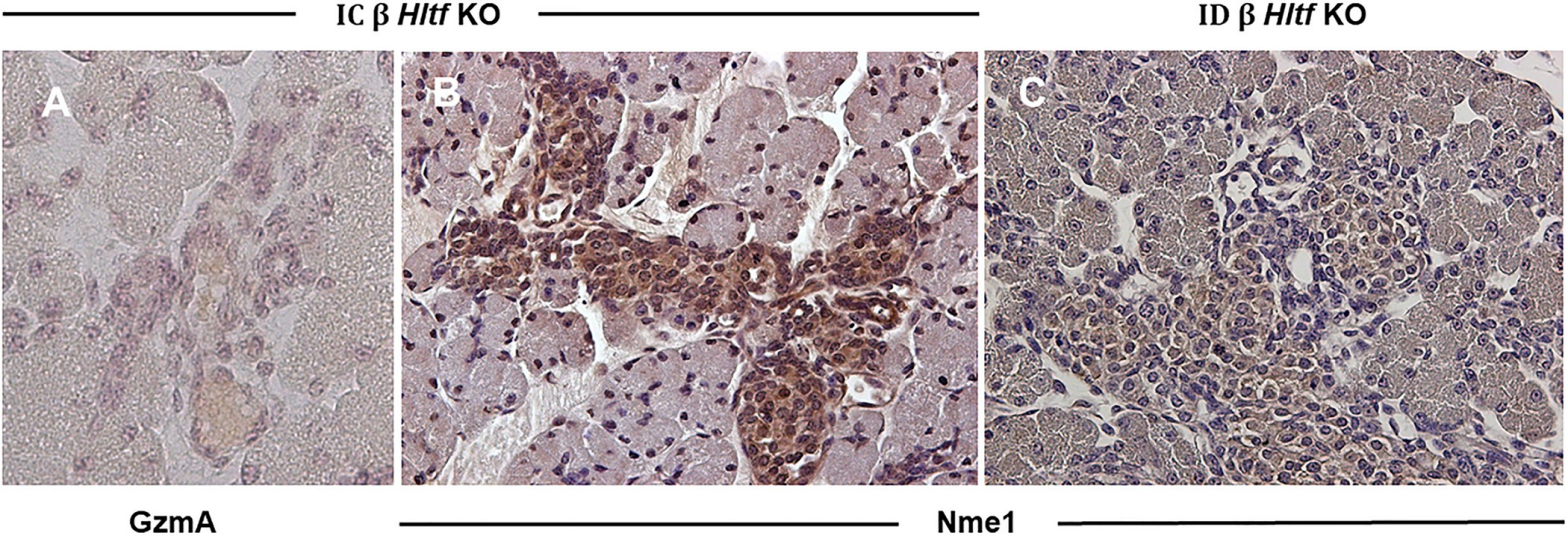
Pancreata from IC and ID β *Hltf* KO mice. Sections from IC β *Hltf* KO mice immunostained for GzmA (A) and Nme1 (B), and ID β *Hltf* KO mice immunostained for Nme1 (C). All sections were counter stained with hematoxylin (40X magnification). GzmA protein expression tracks gene expression in tissue from IC β *Hltf* KO mice (A). In comparison, ID β Hltf *KO* mice on the *Rag2-/-IL2-/-* background have severe lymphocyte developmental impairment (no NK cells). Nme1 protein is immunolocalized to the nuclei of islet β cells in tissue from IC β Hltf KO mice (B) compared to cytoplasmic and perinuclear localization in ID β Hltf KO mice (C). Nme1 lacks a canonical nuclear localization signal and is only translocated to the nucleus as a GzmA-activated DNase.

The expression of GzmA (Fig 7) in the islets of IC β *Hltf* KO mice provided insight to the cell death pathway. GzmA disrupts electron transport by cleaving the electron transport chain complex subunit mitochondrial enzyme NADH dehydrogenase [ubiquinone] iron-sulfur protein 3 (Ndufs3), that has the same level of transcriptional expression (p = 0.116) in pancreata from ID and IC β *Hltf* KO, and activates the nucleosome assembly protein (SET). Activated SET relieves inhibition of the DNase Nme1/Nm23 nucleoside diphosphate kinase 1 (Nme1) that translocates to the nucleus (Fig 7) where it acts together with three prime repair exonuclease 1 (Trex1) that has the same level of transcriptional expression (p = 0.613) in pancreata from ID and IC β *Hltf* KO and causes single-stranded DNA damage. The inflammatory response is amplified by two additional components in the islet microenvironment. The first, decreased transcriptional availability (log2 FC -4.036, p = 0.001) of serine (or cysteine) peptidase inhibitor, clade C (antithrombin), member 1 (SerpinC1) that encodes a serine protease inhibitor antithrombin III (ATIII), the extracellular inhibitor of GzmA. The second, increased transcriptional availability of the complement receptors C3ar1 (log2 FC +1.176, p = 0.023) and C5ar1 (log2 FC +1.310, p = 0.018), that invite crosstalk with Hmgb1 to amplify inflammation.

## Discussion

**Fig 8:**
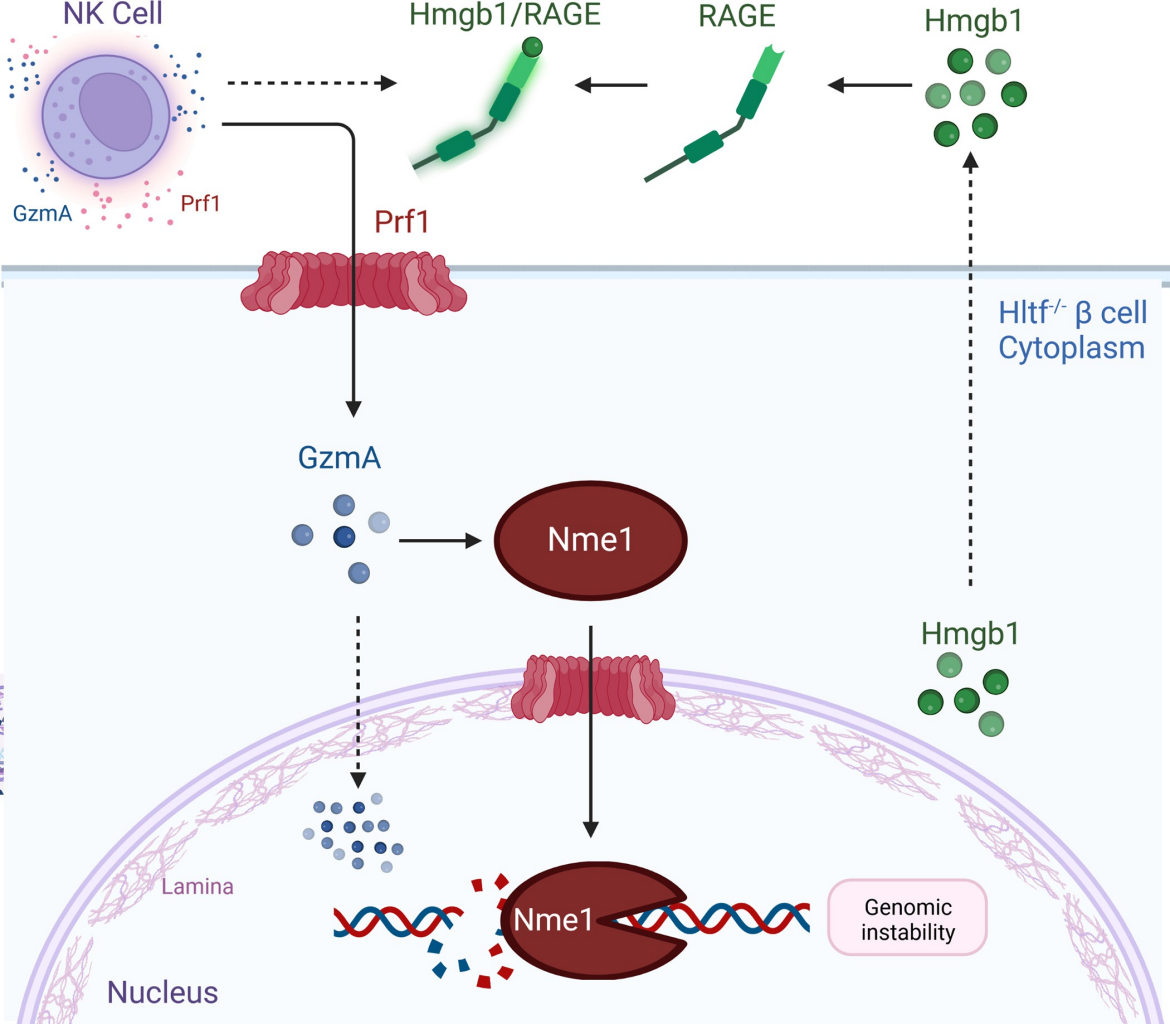
Catastrophic consequences of Hltf-deletion from pancreatic β cells during development. Recruitment of NK cells expressing Prf1-GzmA—triggered by the Hmgb1-RAGE axis in response to impaired DNA-damage repair—perpetuated DNA damage and selective loss of pancreatic β cells. Created with Biorender.com.

Autoimmune destruction of pancreatic β cells is poorly understood, in part, because it is unclear how the β cells and immune cells interact to initiate or perpetuate the process. In this study, ID and IC β *Hltf* KO models allowed us to evaluate the loss of *Hlt*f-facilitated DNA repair in the presence and absence of the immune system *in vivo*. It also allowed us to identify a mechanism whereby the β cells were complicit in their own demise (Fig 8).

ID β *Hltf* KO cells exhibited lower levels of apoptosis in a genetic background that lacks functional receptors for IL-2,-4,-7,-9,-15, and -21, and have severe lymphocyte developmental impairment (deficient T and B cells, no NK cells). In contrast, in the IC β *Hltf* KO model, the activated Hmgb1-Rage axis mediated increased β cell visibility to immune surveillance. We know from the literature that GzmA activates a caspase-independent cell death pathway with morphological features of apoptosis (nuclear fragmentation) via single-stranded DNA damage measurable with TUNEL. Perforin-delivered GzmA to target cell cytoplasm where it activates the endonuclease Nme1 that works in concert with the exonuclease Trex1 to nick DNA [28]. GzmA also traffics to and concentrates in the nucleus. GzmA disrupts the nuclear envelope by cleaving lamins. GzmA further interferes with DNA repair by cleaving and inactivating Parp-1, an ARP-ribosyl transferase enzyme [29], that functions as an early sensor of both single and double stranded DNA damage. GzmA cleaves Parp-1 to separate its DNA binding domain from its catalytic domain. The complete loss of Hltf-Parp1 interaction [30] in the progression and stability of damaged replication forks in conjunction with GzmA-activated Nme1 damage of nuclear DNA explains increased levels and stronger intensities of γH2AX (Fig 5). It also explains why the β cells are unable to repair the DNA damage.

This caspase-independent method of cell death was supported by transcriptional downregulation of the Serpinc1 gene that encodes a serine protease inhibitor of GzmA. No other inhibitors of granzymes have been identified other than this one presumably because GzmA—the most abundant serine protease in killer cell cytoplasmic granules—may require rapid removal from the extracellular milieu. A recent study on the expression of complement receptors, C3ar1 and C5ar1, on human and mouse β cells, indicated they are positive regulators of cell function [31]. This may be true for ID β *Hltf* KO cells. However, increased transcriptional availability of the complement receptors C3ar1 and C5ar1 likely invited crosstalk with Hmgb1 to amplify inflammation in IC β *Hltf* KO cells [32] given the evolutionary conservation of the complement system [14]. Our study is not precisely analogous to neonatal diabetes because IC β *Hltf* KO mice do not have low-birth weights [17] compared to controls (Hltf +/+). However, like neonatal diabetes [33] the study does show that immune attack can start before birth and progress rapidly to complete destruction of insulin producing β cells. Our findings are otherwise compatible with two studies that identified Hltf in the β cell transcriptome [34] and proteome [35] implicating Hltf in pancreatic β cell function.

## Conclusions

HLTF is well known for its role in post-replication repair, and more recently for efficient nucleotide excision repair. To our knowledge, ours is the first *in vivo* experimental system to evaluate the loss of Hltf-facilitated DNA repair in the presence and the absence of the immune system. The results show conclusively that the innate immune system compromises DNA-damage repair and survival when *Hltf* is deleted from pancreatic β cells during development. In as much as disturbances in epigenetics mechanisms can result in developmental dysfunction and disease processes, epigenetic reprogramming has corrective potential.

## Supporting information

S1 Checklist The ARRIVE guidelines 2.0: Author checklist. (PDF)

## References

[1] Achar YJ, Balogh D, Neculai D, Juhasz S, Morocz M, Gali H, et al. Human HLTF mediates postreplication repair by its HIRAN domain-dependent replication fork. Nuclei Acids Res. 2015;43(21):10277–10291. doi: 10.1093/nar/gkv896 PMC4666394 26350214

[2] Li H, Zimmerman SE, Weyemi. Genomic instability and metabolism in cancer. Int Rev Cell Mol Biol. 2021;364:241–265. doi: 10.1016/bs.ircmb.2021.05.004 34507785

[3] Dhont L, Mascaux C, Belayew A. The helicase-like transcription factor (*HLTF*) in cancer: loss of function or oncomorphic conversion of a tumor suppressor. Cell Mol Life Sci. 2016;73(1):129–47. doi: 10.1007/s00018-015-2060-6 26472339 PMC11108516

[4] Motegi A, Liaw H-J, Lee K-Y, Roest HP, Maas A, Wu X, et al. Polyubiquitination of proliferating cell nuclear antigen by HLTF and SHPRH prevents genomic instability from stalled replication forks. Proc Natl Acad Sci U S A. 2008;105(34):12411–6. doi: 10.1073/pnas.0805685105 18719106 PMC2518831

[5] Bai G, Kermi C, Stoy H, Schiltz C, Bacal J, Zaino AM, et al. HLTF promotes fork reversal, limiting replication stress resistance and preventing multiple mechanisms of unrestrained DNA synthesis. Mol Cell. 2020;78(6):1237–1251.e7. doi: 10.1016/j.molcel.2020.04.031 32442397 PMC7305998

[6] Van Toon M, Turkyilmaz Y, Han S, Zhou D, Kim HS, Salas-Armenteros I, et al. Active DNA damage eviction by HLTF stimulates nucleotide excision repair. Mol Cell. 2022;82(7):1343–1358 e8. doi: 10.1016/j.molcel.2022.02.020 35271816 PMC9473497

[7] Collins JA, Schandi CS, Young KK, Vesely J, Willingham MC. Major DNA fragmentation is a late event in apoptosis. J Histochem Cytochem. 1997;45(7):923–934. doi: 10.1177/002215549704500702 9212818

[8] Ragu S, Matos-Rodrigues G, Lopez BS. Replication stress, DNA damage, inflammatory cytokines and innate immune response. Genes (Basel) 2020;11(4):409–434. doi: 10.3390/genes11040409 32283785 PMC7230342

[9] Chen R, Kang R, Tang D. The mechanism of HMGB1 secretion and release. Exp Mol Med. 2022;54(2):91–102. doi: 10.1038/s12276-022-00736-w 35217834 PMC8894452

[10] Zindel J, Kubes P. DAMPS, PAMPS, and LAMPS in immunity and sterile inflammation. Annu Rev Pathol. 2020;15:493–518. doi: 10.1146/annurev-pathmechdis-012419-032847 31675482

[11] Place DE, Kanneganti TD. The innate immune system and cell death in autoinflammatory and autoimmune disease. Curr Opin Immunol. 2020;67:95–105. doi: 10.1016/j.coi.2020.10.013 33242752

[12] Roep BO, Thomaidou S, van Tienhoven R, Zaldumbide A. Type I diabetes mellitus as a disease of the β-cell (do not blame the immune system?). Nat Rev Endocrinol. 2021;17(3):150–161. doi: 10.1038/s41574-020-00443-4 33293704 PMC7722981

[13] Redondo M, Steck A, Pugliese A. Genetics of type 1 diabetes. Pediatr Diabetes. 2018; 19(3):346–353. doi: 10.1111/pedi.12597 29094512 PMC5918237

[14] Faenza I, Blalock WL. Innate immunity: A balance between disease and adaptation to stress. Biomolecules. 2022 May; 12(5): 737–756. doi: 10.3390/biom12050737 35625664 PMC9138980

[15] Helmer RA, Foreman O, Dertien JS, Panchoo M, Bhakta SM, Chilton BS. Role of helicase-like transcription factor (*Hltf*) in the G2/M transition and apoptosis in brain. PLoS One. 2013 Jun 24;8(6):e66799. doi: 10.1371/journal.pone.0066799 23826137 PMC3691323

[16] Helmer RA, Martinez-Zaguilan R, Dertien JS, Fulford C, Foreman O, Peiris V, Bet al. Helicase-like transcription factor (*Hltf*) regulates G2/M transition, Wt1/Gata4/Hif-1a cardiac transcription networks, and collagen biogenesis. PLoS One. 2013;8(11):e80461. doi: 10.1371/journal.pone.0251132 PMC3835564 24278285

[17] Kaur G, Helmer RA, Smith LA, Martinez-Zaguilan R, Dufour JM, Chilton BS. Alternative splicing of helicase-like transcription factor (*Hltf*): intron retention-dependent activation of immune tolerance at the feto-maternal interface. PLoS One. 2018;13(7):e0200211. doi: 10.1371/journal.pone.0200211 29975766 PMC6033450

[18] McClive PJ, Sinclair AH. Rapid DNA extraction and PCR-sexing of mouse embryos. Mol Reprod Dev 2001;60:225–226. doi: 10.1002/mrd.1081 11553922

[19] Azevedo-Pouly ACP, Elgamal OA, Schmittgen TD. RNA isolation from mouse pancreas: a ribonuclease-rich tissue. J Vis Exp 2014;90:51779. doi: 10.3791/51779 25145327 PMC4297470

[20] Griffin M, Abu-El-Haija M, Abu-El-Haija M, Rokhlina T, Uc A. A simplified and versatile method for obtaining high quality RNA from pancreas. Biotechniques. 2012;52(5):332–334. doi: 10.2144/0000113862 22578126 PMC3738267

[21] Stuelsatz P, Keire P, Almuly R, Yablonka-Reuveni Z. A contemporary atlas of the mouse diaphragm: myogenicity, vascularity and the PAX3 connection. J Histochem Cytochem 2012;60(9):638–657. doi: 10.1369/0022155412452417 22723526 PMC3524553

[22] Helmer RA, Kaur G, Smith LA, Chilton BS. Helicase-like transcription factor (*Hltf*) gene-deletion promotes oxidative phosphorylation (OXPHOS) in colorectal tumors of AOM/DSS-treated mice. PLoS One. 2019 Aug 28;14(8):e0221751. doi: 10.1371/journal.pone.0221751 eCollection 2019. 31461471 PMC6713344

[23] Helmer RA, Martinez-Zaguilan R, Kaur G, Smith LA, Dufour JM, Chilton BS. Helicase-like transcription factor-deletion from the tumor microenvironment in a cell line-derived xenograft model of colorectal cancer reprogrammed the human transcriptome-S-nitroso-proteome to promote inflammation and redirect metastasis. PLoS One. 2021 May 19;16(5):e0251132. doi: 10.1371/journal.pone.0251132 34010296 PMC8133447

[24] Lee JY, Ristow M, Lin X, White MF, Magnuson HA, Hennighausen L. RIP-Cre revisited, evidence for impairments of pancreatic beta-cell function. J Biol Chem. 2006;281(5):2649–2653. doi: 10.1074/jbc.M512373200 16326700

[25] Magnuson MA, Osipovich AB. Pancreas-specific Cre driver lines and considerations for their prudent use. Cell Metab. 2013;18(1):9–20. doi: 10.1016/j.cmet.2013.06.011 23823474 PMC3732107

[26] Miller K, Abraham K, Kilimnik G, Jo J, Moka U, Periwal V, et al. Islet formation during the neonatal development in mice. PLoS One. 2009;4(11):e7739 doi: 10.1371/journal.pone.0007739 19893748 PMC2770846

[27] Criner A, Milpied P, Escaliere B, Piperoglou C, Galluso J, Balsamo A, et al. High-dimensional single-cell analysis identifies organ-specific signatures and conserved NK subsets in humans and mice. Immunity. 2018;49(5):971–986.e5. doi: 10.1016/j.immuno.2018.09.009 30413361 PMC6269138

[28] Chowdhury D, Beresford PJ, Zhu P, Zhang D, Sung JS, Demple B, et al. The exonuclease TREX1 is in the SET complex and acts in concert with NM23-H1 to degrade DNA during Granzyme A-mediated cell death. Mol Cell. 2006; 23(1):133–42. doi: 10.1016/j.molcel.2006.06.005 16818237

[29] Zhu P, Martinvalet D, Chowdhury D, Zhang D, Schlesinger A, Liberman J. The cytotoxic T lymphocyte protease granzyme A cleaves and inactivates poly(adenosine 5’-diphosphate-ribose) polymerase-1. Blood. 2009;114(6):1205–16. doi: 10.1182/blood-2008-12-195768 19506301 PMC2723016

[30] Shiu JL, Wu CK, Chang SB, Sun YJ, Chen YJ, Lai CC, et al. The HLTF-PARP1 interaction in the progression and stability of damaged replication forks caused by methyl methanesulfonate. Oncogenesis. 2020;9(12):104. doi: 10.1038/s41389-020-00289-5 33281189 PMC7719709

[31] Atanes P, Ruz-Maldonado I, Pingitore A, Hawkes R, Liu B, Zhao M, et al. C3aR and C5ar1 act as key regulators of human and mouse β-cell function. Cell Mol Life Sci. 2018;7575(4):715–726. doi: 10.1007/s00018-017-2655-1 PMC5769825 28921001

[32] Gaboriaud C, Lorvellec M, Rossi V, Dumestre-Perard C, Thielens NM. Complement system and alarmin HMGB1 crosstalk: for better or worse. Front Immunol. 2022;13: 869720. doi: 10.3389/fimmu.2022.869720 35572583 PMC9095977

[33] Johnson MB, Patel KA, De Franco E, Hagopian W. Killian M, McDonald TJ, et al. Type 1 diabetes can present before the age of 6 months and is characterized by autoimmunity and rapid loss of beta cells. Diabetologia. 2020;63(12):2605–2615. doi: 10.1007/s00125-020-05276-4 33029656 PMC7641942

[34] Bacos K, Gillberg L, Volkov P, Olsson AH, Hansen T, Pedersen O, et al. Blood-based biomarkers of age-associated epigenetic changes in human islets associate with insulin secretion and diabetes. Nature Commun. 2016;7:11089. doi: 10.1038/ncomms11089 27029739 PMC4821875

[35] Horn S, Kirkegaard JS, Hoelper S, Seymour PA, Rescan C, Nielsen JH, et al. Research Resurce: A dual proteomic approach identifies regulated islet proteins during β-cell mass expansion in vivo. Mol Endocrinol. 2016;30(1):133–143. doi: 10.1210/me.2015-1208 26649805 PMC5414659

